# Echo- and B-Type Natriuretic Peptide-Guided Follow-Up versus Symptom-Guided Follow-Up: Comparison of the Outcome in Ambulatory Heart Failure Patients

**DOI:** 10.1155/2018/3139861

**Published:** 2018-09-30

**Authors:** Gani Bajraktari, Nicola Riccardo Pugliese, Andreina D'Agostino, Gian Marco Rosa, Pranvera Ibrahimi, Luan Perçuku, Mario Miccoli, Gian Giacomo Galeotti, Iacopo Fabiani, Roberto Pedrinelli, Michael Henein, Frank L. Dini

**Affiliations:** ^1^Department of Public Health and Clinical Medicine, Umeå University and Heart Centre, Umeå, Sweden; ^2^Clinic of Cardiology, University Clinical Centre of Kosova, Prishtina, Kosovo; ^3^Cardiac, Thoracic and Vascular Department, University of Pisa, Pisa, Italy; ^4^Department of Internal Medicine, University of Genoa, Genoa, Italy; ^5^Department of Clinical and Experimental Medicine, University of Pisa, Pisa, Italy

## Abstract

Recent European Society of Cardiology and American Heart Association/American College of Cardiology Guidelines did not recommend biomarker-guided therapy in the management of heart failure (HF) patients. Combination of echo- and B-type natriuretic peptide (BNP) may be an alternative approach in guiding ambulatory HF management. Our aim was to determine whether a therapy guided by echo markers of left ventricular filling pressure (LVFP), lung ultrasound (LUS) assessment of B-lines, and BNP improves outcomes of HF patients. Consecutive outpatients with LV ejection fraction (EF) ≤ 50% have been prospectively enrolled. In Group I (*n*=224), follow-up was guided by echo and BNP with the goal of achieving E-wave deceleration time (EDT) ≥ 150 ms, tissue Doppler index *E*/*e*′ < 13, B-line numbers < 15, and BNP ≤ 125 pg/ml or decrease >30%; in Group II (*n*=293), follow-up was clinically guided, while the remaining 277 patients (Group III) did not receive any dedicated follow-up. At 60 months, survival was 88% in Group I compared to 75% in Group II and 54% in Group III (*χ*^2^ 53.5; *p* < 0.0001). Survival curves exhibited statistically significant differences using Mantel–Cox analysis. The number needed to treat to spare one death was 7.9 (Group I versus Group II) and 3.8 (Group I versus Group III). At multivariate Cox regression analyses, major predictors of all-cause mortality were follow-up *E*/*e*′ (HR: 1.05; *p*=0.0038) and BNP >125 pg/ml or decrease ≤30% (HR: 4.90; *p*=0.0054), while BNP > 125 pg/ml or decrease ≤30% and B-line numbers ≥15 were associated with the combined end point of death and HF hospitalization. Evidence-based HF treatment guided by serum biomarkers and ultrasound with the goal of reducing elevated BNP and LVFP, and resolving pulmonary congestion was associated with better clinical outcomes and can be valuable in guiding ambulatory HF management.

## 1. Introduction

Historically, the response to unloading therapy of elevated LV filling pressure (LVFP) has been identified as an important mode to risk stratify patients with heart failure (HF) who underwent right heart catheterization [1]. Recent advances support the value natriuretic peptide (NP) circulating level assessment [2] and pulmonary artery pressure monitoring [3, 4] as tools for scrutinizing patients with impending clinically overt HF. It is well known that similar hemodynamic information to that provided by the invasive standard can be obtained noninvasively by Doppler echocardiography; particularly, an E-wave deceleration time (EDT) < 150 ms and a ratio of E/averaged myocardial early velocity (averaged *E*/*e*′) ≥ 13 have been found to reliably reflect increased LVFP and worse prognosis [5–7], whereas reversal of these alterations has been associated with more favorable outcomes [8, 9]. Combining lung ultrasound (LUS) assessment of B-lines with EDT and *E*/*e*′ may increase the diagnostic accuracy in estimating LVFP and in identifying pulmonary congestion [10]. Therefore, the combination of Doppler echocardiographic markers of LVFP, LUS, and NPs may be potentially valuable in guiding ambulatory HF management, since they can be useful in distinguishing stable patients from those at high risk of decompensation, optimizing treatment, reducing hospitalization, and consequently improving prognosis [11, 12]. To investigate this concept, we compared the outcome of ambulatory patients with chronic HF and reduced or mildly reduced LV ejection fraction (EF) divided according to follow-up strategies: echo Doppler signs of elevated LVFP, LUS, and B-type natriuretic peptide (BNP)-directed follow-up (Group I); symptom-guided follow-up (Group II); and no organized follow-up for cardiovascular care (Group III).

## 2. Materials and Methods

This observational study included consecutive ambulatory patients with chronic HF and reduced or mildly reduced LVEF. All patients underwent baseline evaluation by an experienced cardiologist, including blood tests and 12-lead ECG. Patients' functional status was determined according to the classification of the New York Heart Association (NYHA). At baseline, complete M-mode, two-dimensional, and Doppler echocardiogram was carried out in all study patients according to the recommendations of the European Association of Echocardiography/American Association of Echocardiography [13].

**Figure 1:**
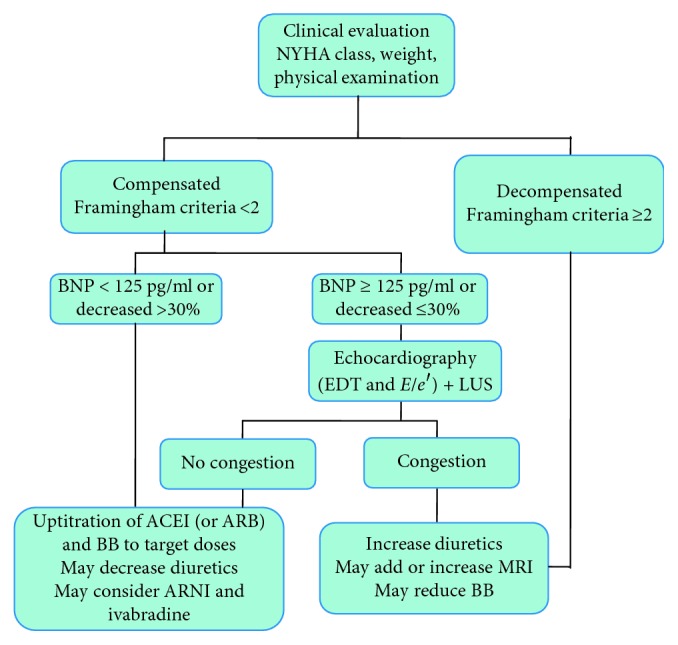
Predefined titration protocol. ACEI: angiotensin-converting enzyme inhibitors; ARB: angiotensin receptor blockers; ARNI: angiotensin receptor neprilysin inhibitors; BB: beta-blockers; BNP: B-type natriuretic peptide; EDT: E-wave deceleration time; *E*/*e*′: ratio of *E*/averaged myocardial early velocity; MRI: mineralocorticoid receptors inhibitors; LUS: lung ultrasound.

The study population comprised of patients enrolled between 2001 and 2016:

1. Group I: 224 patients from the dedicated outpatient ambulatory of the Cardiovascular Division of the Cardiovascular and Thoracic Department of the University Hospital of Pisa, whose management was directed according to the presence of echo Doppler signs of elevated LVFP, LUS, and BNP levels. At each visit, patients were clinically assessed (history, clinical examination, NYHA class, and weight) and were classified according to the Framingham criteria [14]. Blood was drawn for BNP assay and measure of renal function. An echocardiogram was carried out for assessing LVFP, as an adjunct to the physical examination in case of inconclusive signs and symptoms and BNP >125 pg/ml [15]. The following Doppler echocardiographic findings were considered as surrogate markers of increased LVFP: *E*/*e*′ ≥ 13 and EDT < 150 ms. The echographic examination of the lungs for B-lines assessment was eventually performed, and patients were classified according to B-line numbers ≥15 and <15 [16]. Therapeutic interventions were made according to a titration protocol (Figure 1). Our aim was to optimize treatment with neurohormonal drugs in compensated HF patients and to lower elevated BNP and LVFP and resolve pulmonary congestion in decompensated HF patients. The frequencies of follow-up visits were decided on the basis of physical, biochemical, and echographic findings.
2. Group II: 293 consecutive patients from the dedicated outpatient ambulatory of the HF Unit of the Cardiovascular and Thoracic Department of the University Hospital of Pisa, whose management was clinically guided according to a standardized protocol that included medical history, clinical examination, blood tests, and 12-lead ECG. These patients underwent an echocardiographic examination at baseline and again if clinical changes occurred. In this group, NPs were not systematically evaluated during follow-up. The frequencies of follow-up visits were based on clinical judgment.
3. Group III: 277 consecutive ambulatory patients from the Cardiovascular Division of Santa Chiara Hospital of Pisa. Patients of this group did not receive a specific follow-up for cardiovascular care and were mostly followed by their family physicians who were consulted in case of relapses.

The study was approved by the local institutional review boards. All patients gave written informed consent. The study was conducted in accordance with institutional policies, national legal requirements, and the revised Helsinki Declaration.

Information on patients' outcomes was obtained through clinical visits, personal communication with general physicians, and telephone interviews with patients and relatives, by trained personnel. The primary study end point was all-cause mortality, and the secondary end point was the combination of death and hospitalization for worsening HF.

Data are summarized using frequencies (percentages) for categorical variables and mean ± standard deviation for continuous variables or median interquartile (IRQ) ranges, when appropriate. Differences between the three groups were tested by analysis of variance (ANOVA) using Bonferroni test. Demographic, clinical, and echo variables were evaluated for the end points in a univariable Cox proportional hazard model (95% confidence intervals). Variables showing significant association with outcome (*p* < 0.1) were included in the multivariable Cox model to determine the ones independently related to prognosis. The cumulative survival probability was explored using the Kaplan–Meier method, followed by the log-rank test. Differences of survival curves were tested with Mantel–Cox statistic log-rank analysis. A *p* value <0.05 was considered statistically significant. All analyses were performed using the IBM SPSS Software Package version 17.0.1.

## 3. Results

Table 1 outlines the baseline demographic, clinical, and echocardiographic characteristics of the three groups. In Group I, baseline BNP was 290 pg/ml (IRQ: 163–571) and follow-up BNP at 6 ± 4 months was 155 pg/ml (IRQ: 58–333) (Mann–Whitney *p* < 0.0001).

**Figure 2:**
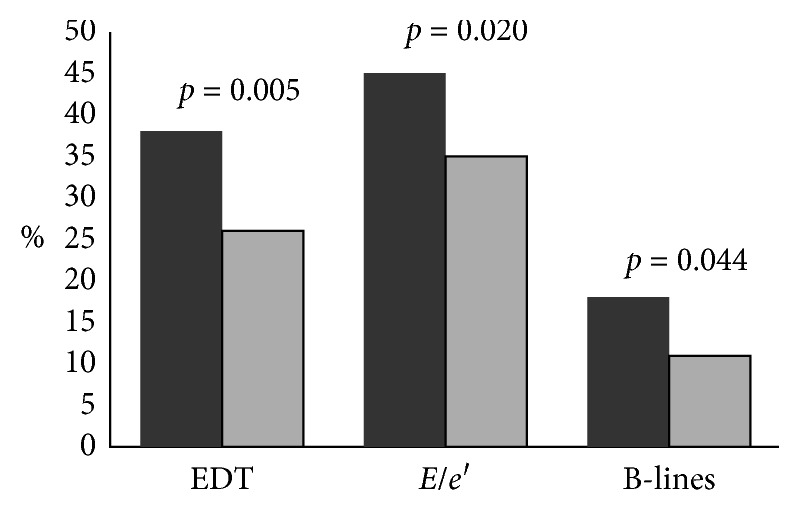
Frequencies of patients with an E-wave deceleration time (EDT) < 150 ms, a ratio of E/averaged myocardial early velocity (averaged *E*/*e*′) ≥ 13, and presence of ≥15 B-lines at lung ultrasound. Comparison between baseline (dark gray bar) and follow-up (light gray bar).

Figure 2 shows percentages of patients of Group I that exhibited echo Doppler markers of raised LVFP and LUS B-line numbers ≥15 at baseline and after 6-month follow-up. Patients who had low BNP levels (≤125 pg/ml) at 6-month follow-up were 23%, 45% of patients had a 6-month decrease >30% from elevated BNP baseline levels, and 34% had elevated BNP levels (>125 pg/ml) at follow-up without any 6-month decrease >30% from their baseline value.

The median follow-up duration was 36 months (IRQ: 17–58) and was comparable in the three groups. Forty-nine events (23 deaths and 26 hospitalizations related to HF) occurred in Group I, 106 in Group II (66 deaths and 40 hospitalizations due to HF), and 152 in Group III (100 deaths and 52 hospitalizations due to HF), respectively. The mean number of follow-up visits/year was 2.5 ± 1.7 in Group I patients and 2.8 ± 1.7 in Group II patients.

**Figure 3:**
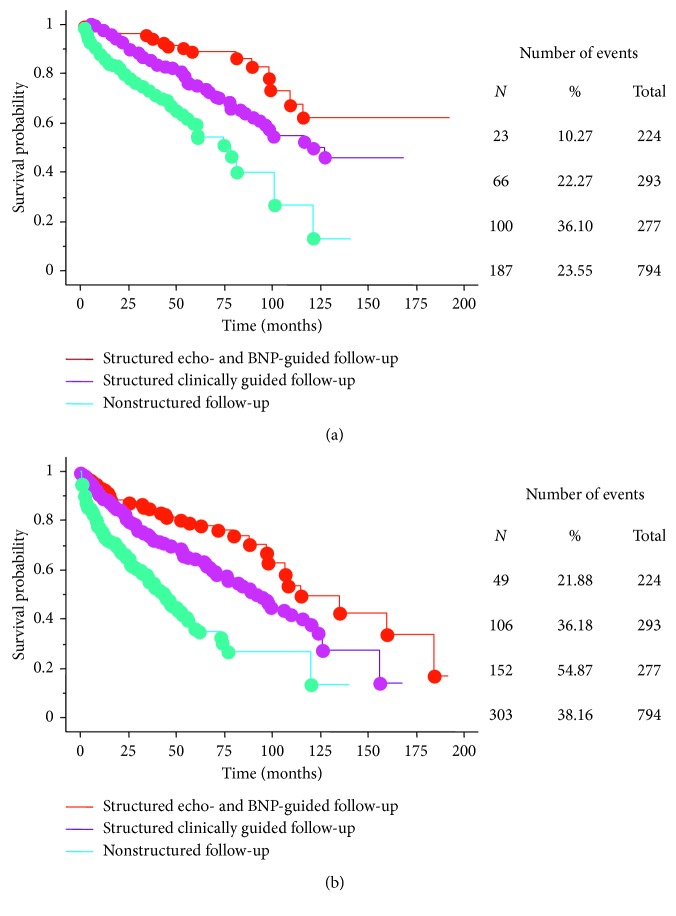
(a) Survival free from all-cause mortality in patients of the echo- and BNP-guided follow-up, in patients of the clinically driven follow-up, and in those who received no specific follow-up care (*X*^2^ 53.5; *p* < 0.0001). (b) Survival curves for the combined end point of death or hospitalization for worsening heart failure in echo- and BNP-guided and symptom-guided groups and in patients who received no specific follow-up care (*X*^2^ 72.4; *p* < 0.0001). When compared to patients of the symptom-guided group and those who did not receive any organized follow-up care, life was estimated to be prolonged by an average of 2.5 years and 4 years, respectively, by the echo- and BNP-guided strategy. Similar results were attained with the event-free life gain.

At 60-month follow-up, survival free from all-cause mortality was 88% in Group I patients, 75% in Group II patients, and 54% in Group III patients (*X*^*2*^ 53.5; *p* < 0.0001). Mantel–Cox analysis showed significant differences between Group I and Group II patients (*X*^*2*^ 8.8; *p* = 0.003), between Group I and Group III patients (*X*^*2*^ 46.2; *p* < 0.0001), and between Group II and Group III patients (*X*^*2*^ 21.5; *p* < 0.0001) (Figure 3). The strategy based on echo and BNP improved the probability of survival by 12.5% versus the symptom-guided follow-up and by 40.7% versus the nonspecific follow-up care. The number needed to treat to spare one death was 8 (Group I versus Group II) and 4 (Group I versus Group III).

Survival free from all-cause mortality and HF-related hospitalization was 76% in Group I patients versus 64% in Group II patients and 36% of Group III patients (*X*^*2*^ 72.4; *p* < 0.0001). Mantel–Cox analysis showed statistically significant differences between Group I and Group II patients (*X*^*2*^ 8.1; *p*=0.004), between Group I and Group III patients (*X*^*2*^ 59.2; *p* < 0.0001), and between Group II and Group III patients (*X*^*2*^ 34.1; *p* < 0.0001) (Figure 3). The number needed to spare one event was 7 (Group I versus Group II) and 3 (Group I versus Group III).

The impact of Group I strategy on survival was evaluated by Cox analysis. Primary end point (all-cause mortality) results are shown in Table 2. Several univariate predictors of all-cause mortality were identified. However, only follow-up BNP >125 pg/ml and decrease ≤30%, averaged *E*/*e*′, and LVEF remained independently associated with outcome on multivariate analysis. The combined end point of death and HF hospitalization was associated with a number of variables on univariate analysis. Multivariate predictors of the combined end point are shown in Table 3. Particularly, BNP >125 pg/ml and decrease ≤30%, presence of ≥15 chest B-lines, and LVEF at follow-up were independently associated with the outcome.

As far as changes in medication during the observation period are concerned, the use of loop diuretics decreased by 11% in Group I and increased by 4.2% in Group II, beta-blocker use increased by 21.3% in Group I and by 0.9% in Group II, the use of ACE inhibitors or angiotensin receptor antagonists increased by 1.5% in Group I and decreased by 10.5% in Group II, and the use of mineralocorticoid receptor inhibitors decreased by 8.6% in Group I and increased by 1.8% in Group II. Eighty-three patients of Group I, who had been previously treated with ACE inhibitors or angiotensin receptor antagonists, received sacubitril/valsartan that was titrated to the maximal tolerated dose. At the end of follow-up, 68 patients of Group I and 53 patients of Group II had a cardioverter defibrillator implanted, whereas 40 and 44, respectively, were submitted to cardiac resynchronization therapy.

## 4. Discussion

The main finding of our study is that follow-up care directed by echo and BNP improved survival in ambulatory patients with HF and reduced or mildly reduced LVEF with respect to patients followed by conventional clinical parameters and those who received no dedicated follow-up. Tissue Doppler index *E*/*e*′ was the best predictor of survival in patients of the echo- and BNP-guided group. In addition, presence of pulmonary congestion as assessed by the increased number of B-lines at LUS was another clinically measurable predictor of outcome.

After hospital discharge, patients with HF remain at high risk of mortality and rehospitalization, particularly during the first few weeks [17, 18]. In most countries, only a minority of patients gain access to a dedicated outpatient clinic, which can provide specific follow-up visits for cardiovascular care [19].

Care management programs for HF have traditionally focused on patients with chronic HF at high risk of decompensation detected during outpatient follow-up. The growing pressure from hospital readmissions in HF patients is shifting the focus of interest from traditionally symptom-guided care to a more specific patient-centered follow-up care based on clinical findings, NPs, and echo [20, 21].

As it is known, NP-guided therapy has not received an endorsement as the recommended approach for HF management by the American and European society guidelines [22, 23]. In the recent GUIDE-IT study, where HF patients with LVEF ≤ 40% were randomized to either an NT-proBNP-guided strategy or usual care, no significant differences have been reported between the NP-guided arm and the usual care arm in reducing time to first hospitalization or cardiovascular mortality. However, a major limitation of GUIDE-IT was that a single target value of NT-proBNP < 1,000 pg/mL was utilized [24].

Regardless of the etiology, increases in LVFP and pulmonary congestion are necessary prerequisites to decompensation. In addition to echo Doppler variables that allow estimation of LVFP, that is, *E*/*e*′ and EDT, LUS may be utilized for the prediction of decompensated HF [25]. The ability to distinguish stable from unstable HF patients may be refined by the concomitant assessment of NP circulating levels and their variations over time [26].

In the last years, LUS has been proposed for the evaluation of pulmonary congestion, through the assessment of B-lines. Quantification of B-lines has been shown to be useful for the diagnosis, monitoring, and risk stratification of patients with known or suspected acute HF [27] and may add to the assessment of hemodynamic congestion by echo Doppler parameters and NPs also in an outpatient setting [12, 28]. The echo Doppler estimation of LVFP by *E*/*e*′ and NPs is somewhat correlated with pulmonary tissue water in large populations, but may provide different information in the single patient [29].

Beyond the prognostic value of reducing NPs to less than a specific numeric value, changes in NP concentrations over time may help to better stratify the risk [30]. This is the reason why, rather than simply rely on fixed BNP values, we have used a combination of follow-up BNP <125 pg/ml and decrease >30% as targets of therapy [31].

Our results support the use of Doppler echocardiographic signs of raised LVFP and LUS with BNP in the follow-up of HF outpatients with reduced or mildly reduced EF. The improvement in clinical outcome of ambulatory patients with systolic HF who underwent follow-up evaluations that included echocardiography, LUS, and the assessment NPs can be attributed to prevention of clinically overt pulmonary congestion, refractoriness to loop diuretics, and a better titration of cardiovascular drugs. Moreover, repeated echocardiograms during follow-up may be valuable for the earlier identification of candidates to surgical and percutaneous valve interventions, cardiac resynchronization therapy, and cardioverter defibrillator implantation. Finally, drug treatments with newer pharmacological entities, such as sacubitril/valsartan, that have been recently introduced in the clinical scenario, may importantly contribute to improve patients' prognosis and decrease the occurrence of worsening HF [32].

Our study has several limitations. Data were collected retrospectively, and follow-up visits were planned and made according to different protocols. Despite the recent development of more sophisticated echocardiographic techniques, for example, speckle tracking spectral Doppler [33], echo Doppler markers of raised LVFP are well validated and universally used.

Due to its simple application, repeatability, and open access in daily HF management, assessment of markers of raised LVFP and LUS in association with repeated determination of NP circulating levels appears valuable to guide therapy of ambulatory HF patients. Nevertheless, further work is necessary to validate our findings and this investigation must be considered only a hypothesis-generating study whose results need to be confirmed by a prospective randomized trial. The *E*/*e*′, which is one of the parameters used to determine diastolic dysfunction as per the most recent guidelines, should be followed over time in future studies.

## Figures and Tables

**Table 1:** Demographic, clinical, and echocardiographic baseline characteristics of the study groups.

Variable	Group I (*n*=224)	Group II (*n*=293)	Group III (*n*=277)	*p* value
Age (years)	67 ± 12	68 ± 11++	70 ± 10‡‡	<0.0001
Follow-up (months)	35 (12–66)	36 (19–65)	39 (20–55)	0.057
Male (%)	78	80	80	0.72
Heart rate (beats/min)	75 ± 14	76 ± 15	75 ± 15	0.22
Ischemic CM (%)	50	59	62‡‡	0.024
eGFR <60 ml/m^2^ (%)	33	40	36	0.27
DM (%)	30	24	22‡	0.13
History of hypertension	46	49	45	0.59
Atrial fibrillation (%)	19	16	19	0.54
NYHA class > II (%)	24	27	31	0.18
Systolic BP (mmHg)	123 ± 18^*∗∗*^	133 ± 20++	124 ± 17‡‡	<0.0001
Diastolic BP (mmHg)	74 ± 10^*∗∗*^	76 ± 15++	73 ± 9‡‡	<0.0001
LV EDVi (ml/m^2^)	100 ± 33^*∗∗*^	86 ± 33++	109 ± 32‡‡	<0.0001
LV ESVi (ml/m^2^)	68 ± 29^*∗∗*^	59 ± 31++	75 ± 29‡‡	<0.0001
LVEF (%)	33 ± 8	34 ± 9	33 ± 8‡	0.070
Mitral regurgitation† (%)	28	29	37	0.039
EDT (msec)	179 ± 58^*∗∗*^	212 ± 62	159 ± 53‡‡	<0.0001
Loop diuretics (%)	82	82	86	0.27
Beta-blockers (%)	69^*∗*^	79++	66	0.016
ACE inhibitors or angiotensin receptor inhibitors (%)	89	86++	76‡‡	0.003
Mineralocorticoid receptor inhibitors (%)	62	58	51	0.29
CRT (%)	9	8	8	0.90
ICD (%)	17	11	12	0.071

CM: cardiomyopathy; eGFR: estimated glomerular filtration rate; DM: diabetes; NYHA: New York Heart Association; BP: blood pressure; LV: left ventricular; EDVi: end-diastolic volume index; ESVi: end-systolic volume index; LVEF: left ventricular ejection fraction; EDT: E-wave deceleration time; CRT: cardiac resynchronization therapy; ICD: implantable cardioverter defibrillator.^*∗*^: *p* < 0.05,^*∗∗*^: *p* < 0.01 echo- and BNP-guided group versus symptom-guided group; +: *p* < 0.05, ++: *p* < 0.01 symptom-guided group versus no follow-up; ‡: *p* < 0.05, ‡‡: *p* < 0.01 echo- and BNP-guided group versus no follow-up.

**Table 2:** Univariate and multivariate predictors of all-cause mortality among 224 patients of the BNP- and echo-guided group.

Variable	Univariate		Multivariate	
	HR (95% CI)	*p*-value	HR (95% CI)	*p*-value
Age	1.03 (1.00, 1.05)	0.043		
Male	0.98 (0.51, 1.97)	0.98		
Heart rate	1.01 (1.00, 1.03)	0.11		
Ischemic CM	1.23 (0.88, 1.72)	0.29		
Diabetes	1.98 (1.13, 3.45)	0.021		
Hypertension	0.97 (0.55, 1.73)	0.93		
Atrial fibrillation	1.39 (0.68, 2.85)	0.38		
NYHA class	2.38 (1.67, 3.38)	<0.0001		
Systolic BP	0.97 (0.95, 0.99)	0.0022		
Diastolic BP	0.96 (0.93, 0.99)	0.0032		
eGFR	0.98 (0.97, 0.99)	0.0040		
BNP	1.00 (1.00, 1.00)	<0.0001		
BNP >125 pg/ml or decrease ≤30%	5.74 (2.97, 11.07)	<0.0001	1.55 (1.35, 1.77)	0.0038
LVEF	0.89 (0.85, 0.92)	<0.0001	0.94 (0.89, 1.00)	0.051
Mitral regurgitation	3.58 (3.00, 6.38)	<0.0001		
EDT	0.99 (0.98, 0.99)	<0.0001		
*E*/*e*′ averaged	1.06 (1.04, 1.08)	<0.0001	1.05 (1.02, 1.08)	0.0054
B-lines ≥ 15	7.32 (4.13, 12.96)	<0.0001		

HR: hazard ratio. For other abbreviations, see Table 1.

**Table 3:** Univariate and multivariate predictors of the combined end point among 224 patients of the BNP- and echo-guided group.

Variable	Univariate		Multivariate	
	HR (95% CI)	*p*-value	HR (95% CI)	*p*-value
Age	1.03 (1.00, 1.05)	0.071		
Male	0.56 (0.24, 1.37)	0.56		
Heart rate	1.02 (1.01, 1.04)	0.026		
Ischemic CM	1.14 (0.67, 1.94)	0.64		
Diabetes	3.14 (1.44, 6.80)	0.0057		
Hypertension	1.41 (0.62, 3.23)	0.42		
Atrial fibrillation	1.62 (0.8, 4.55)	0.38		
NYHA class	2.29 (1.37, 3.84)	0.0016		
Systolic BP	0.98 (0.96, 1.00)	0.14		
Diastolic BP	0.95 (0.91, 0.99)	0.0079		
eGFR	0.98 (0.96, 0.99)	0.0073		
BNP	1.00 (1.00, 1.00)	0.0033		
BNP >125 pg/ml or decrease ≤30%	8.52 (2.89, 25.16)	<0.0001	2.48 (1.14, 5.42)	0.023
LVEF	0.88 (0.84, 0.94)	<0.0001	0.95 (0.91, 1.00)	0.052
Mitral regurgitation	3.58 (3.00, 6.38)	<0.0001		
EDT	0.98 (0.97, 0.99)	<0.0001		
*E*/*e*′ averaged	1.06 (1.04, 1.09)	<0.0001		
B-lines ≥ 15	5.64 (2.47, 12.83)	<0.0001	2.62 (1.26, 5.42)	0.0099

For abbreviations, see Table 2.

## Data Availability

The datasets generated during and/or analysed during the current study are available from the corresponding author on reasonable request.
